# Colorectal Cancer Diagnosis through Breath Test Using a Portable Breath Analyzer—Preliminary Data

**DOI:** 10.3390/s24072343

**Published:** 2024-04-07

**Authors:** Arcangelo Picciariello, Agnese Dezi, Leonardo Vincenti, Marcello Giuseppe Spampinato, Wenzhe Zang, Pamela Riahi, Jared Scott, Ruchi Sharma, Xudong Fan, Donato F. Altomare

**Affiliations:** 1 Department of Experimental Medicine, University of Salento, 73100 Lecce, Italy; 2 Department of Precision and Regenerative Medicine and Ionian Area and Interdepartmental Research Center for Pelvic Floor Diseases (CIRPAP), University Aldo Moro of Bari, 70124 Bari, Italy; 3 Surgical Unit, IRCCS de Bellis, Castellana Grotte, 70013 Bari, Italy; dr.leonardo.vincenti@gmail.com; 4 Department of Surgery, “Vito Fazzi” Hospital, Piazza Filippo Muratore, 73100 Lecce, Italy; marcello.spampinato@gmail.com; 5 Biomedical Engineering Department, University of Michigan, 1101 Beal Ave., Ann Arbor, MI 48109, USA; wenzhez@umich.edu (W.Z.); jaredmsc@umich.edu (J.S.); rusharma@umich.edu (R.S.); xsfan@umich.edu (X.F.)

**Keywords:** colorectal cancer screening, volatile organic compounds, micro GC, breath biopsy

## Abstract

Screening methods available for colorectal cancer (CRC) to date are burdened by poor reliability and low patient adherence and compliance. An altered pattern of volatile organic compounds (VOCs) in exhaled breath has been proposed as a non-invasive potential diagnostic tool for distinguishing CRC patients from healthy controls (HC). The aim of this study was to evaluate the reliability of an innovative portable device containing a micro-gas chromatograph in enabling rapid, on-site CRC diagnosis through analysis of patients’ exhaled breath. In this prospective trial, breath samples were collected in a tertiary referral center of colorectal surgery, and analysis of the chromatograms was performed by the Biomedical Engineering Department. The breath of patients with CRC and HC was collected into Tedlar bags through a Nafion filter and mouthpiece with a one-way valve. The breath samples were analyzed by an automated portable gas chromatography device. Relevant volatile biomarkers and discriminant chromatographic peaks were identified through machine learning, linear discriminant analysis and principal component analysis. A total of 68 subjects, 36 patients affected by histologically proven CRC with no evidence of metastases and 32 HC with negative colonoscopies, were enrolled. After testing a training set (18 CRC and 18 HC) and a testing set (18 CRC and 14 HC), an overall specificity of 87.5%, sensitivity of 94.4% and accuracy of 91.2% in identifying CRC patients was found based on three VOCs. Breath biopsy may represent a promising non-invasive method of discriminating CRC patients from HC.

## 1. Introduction

Mortality for colorectal cancer is still a major concern for the health system in Western countries [1], but it can be significantly reduced by early diagnosis [2,3]. In fact, one of the major challenges to modern oncology is the early diagnosis of cancer by an effective screening test.

Colorectal cancer screening based on fecal occult blood test has been demonstrated to be able to save lives [4]; however, it is burdened by low patient compliance due to the unpleasant fecal manipulation and limited by unsatisfactory reliability [5].

Nowadays, with precision medicine becoming more widely accessible [6], the time has come to change the way colorectal cancer screening takes place, looking for new, reliable, non-expensive and well-accepted screening tools. Recently, the study of the molecular components of breath has been applied to the early diagnosis and follow-up of several diseases including COVID-19 [7,8], opening the way to a new branch of metabolomics, such as breathomics [9].

Volatile organic compounds (VOCs) are present in various excreted biological materials (urine, blood, faeces and breath), and their analysis offers a possibility for cancer screening [10,11,12,13].

Endogenous breath VOCs can arise from metabolic activity and can originate anywhere in the body.

In patients with cancer, the increased prevalence of reactive oxygen species within cancer cells leads to (per)oxygenation of cell-membrane-based polyunsaturated fatty acids, resulting in an alteration in VOCs produced [14,15]. VOCs reach the pulmonary alveoli through the circulatory system and are exhaled, allowing their objective measurement. VOCs produced by human metabolism are mainly represented by benzene, alkanes and aldehydes (or their derivatives), and several studies have demonstrated that specific VOCs are associated with specific types of cancer, such as lung, breast, hepatocellular carcinoma, melanoma, mesothelioma and gastric cancer [16,17,18,19].

Previous studies from our group [12,20] have demonstrated that colorectal cancer patients have an altered pattern of VOCs in exhaled breath compared to healthy subjects, suggesting that breath analysis could be used as a potential non-invasive diagnostic tool for the detection of colorectal cancer.

However, the technology available is complex, time-consuming and unable to give an immediate response.

This study aims to evaluate the reliability of an innovative, portable device containing a micro-gas chromatograph to enable rapid colorectal cancer diagnosis.

## 2. Methods

This prospective trial was carried out between July 2021 and January 2023 in a tertiary referral center of colorectal surgery at University “Aldo Moro” of Bari. Data analysis and interpretation were performed at Michigan University (Ann Arbor USA). The protocol was approved by the local independent Ethics Committee.

Patients’ age, BMI, comorbidities, drug intake, family history of colorectal cancer and oncological biomarkers (CEA, Ca 19.9) were prospectively recorded on an Excel database.

Inclusion criteria for CRC patients was histologically proven colorectal cancer with no clinical evidence of metastases before surgery, while healthy controls were selected among patients attending the coloproctological outpatient for constipation or rectal bleeding who had a negative colonoscopy within the last 2 years. Intellectually disabled subjects and patients affected by IBD, major systemic diseases (liver, kidney, heart and respiratory failure), previous or concomitant malignancy and those having had a mechanical bowel preparation in the last 2 weeks were excluded. Written informed consent was obtained before entering the study.

## 3. Breath Analysis

The portable gas chromatograph (GC) system used in this study has been reported in previous works published by our study group [7,21]. Briefly, the portable GC consists of a sampling module and an analyzing module. The sampling module is made up of a sampling tube, a thermal desorption tube (5 cm long copper tube; inside diameter (i.d.): 1 mm) loaded with both Carbopack X and B granules (10 mg each, 60–80 mesh, Sigma Aldrich, St Louis, MI, USA), valves and a pump. The analyzing module comprises a stainless steel thermal injector (SSTI) loaded with Carbopack X and B (60–80 mesh), one 10 m long non-polar DB-5 ms column (250 μm × 0.25 μm, Agilent J&W Scientific, Folsom, CA, USA) and a micro-photoionization detector. The device weighs less than 3 kg and is housed in a customized small plastic case.

**Figure 1:**
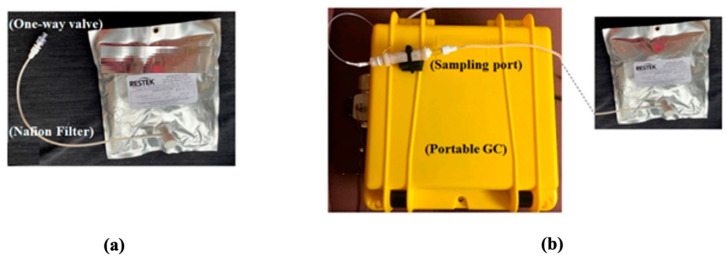
(**a**) The setup shown is used in another study to collect breath in a 1 L bag via a one-way mouthpiece and a Nafion filter. The present study used a similar setup; only the 1 L bag was replaced with a 5 L bag. (**b**) Photo of the portable GC showing that the bag was connected to the sampling port of the device. Image courtesy [22].

Breath collection and analysis were conducted in the same environment and same room by the same operator (AD) on all in-patients admitted to the hospital for colorectal cancer treatment and on patients attending the outpatient clinic for constipation or hemorrhoids (healthy controls), after obtaining written informed consent. The participants were asked to exhale 1 to 2 L of breath into a 5 L bag via a one-way mouthpiece and a Nafion filter (Biopac Systems Inc., Goleta, CA, USA) for moisture removal (Figure 1a). Breath analysis took place either in situ within 18 h of breath collection. During breath analysis, the multi-layer foil bag from Restek (Restek Corporation, Bellefonte, PA, USA) was connected to the sampling port of the device (Figure 1b), and 350 mL of breath was sampled from the bag into the GC for analysis. The GC operation was controlled using LabView via a laptop. During sampling, the VOCs were trapped by the thermal desorption tube. Then, the tube was heated to 300 °C in 1 min and maintained at 300 °C for 4 min to transfer the trapped VOCs to the SSTI. Finally, the SSTI was heated to 250 °C in 0.3 s and maintained at 250 °C for 5 s to inject the VOC pulse into the separation column. The column temperature started from and stayed at 25 °C for 2 min and was then heated to 80 °C with a ramping rate of 10 °C/min. Then, the temperature was raised to 120 °C with a ramping rate of 40 °C/min and held at 120 °C for 1 min. The flow rate of the carrier gas (helium) was 2 mL/min. Assay time included 5 min for breath sampling time from the Tedlar bag (70 mL/min), 5 min of desorption/transfer time, 10 min of chromatographic separation time and 10 min of GC system cleaning time, for a total of 30 min assay time. After breath analysis, consumables (bag and one-way mouthpiece) were disposed of.

**Figure 2:**
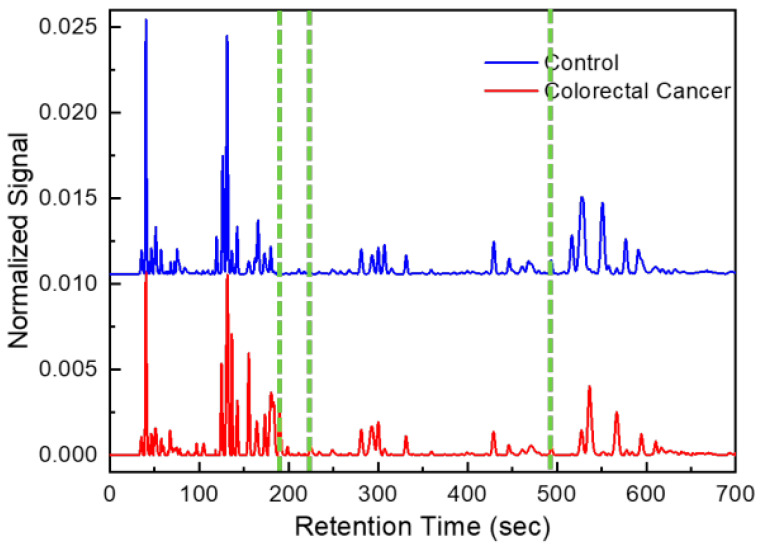
Example of chromatograms obtained from CRC patient and healthy control. Green lines correspond to peaks of discriminant volatile organic compounds (form left to right: hexane 2,4-dimethyl, heptane 2,5-dimethyl and hexane 2,2,5,5-tertramethyl).

One chromatogram, a series of time intervals and detector signal intensities were generated for each GC analysis of a breath sample (Figure 2). After patient breath analysis, chromatograms were pre-processed (such as baseline correction and de-noising) [21,22].

The chromatograms obtained were anonymized and marked with a serial number and sent without sensitive data to Michigan University for data analysis.

## 4. Statistical Analysis

The data analysis pipeline was developed in-house and was implemented using Matlab, version R2021a (MathWorks). Linear discriminant analysis (LDA) and principal component analysis (PCA), already tested and validated in our earlier studies on other pathologies [1,2,3,4], were used for data set dimensionality reduction, biomarker selection and statistical analyses [7,21,22]. Details of the biomarker discovery algorithm have been previously reported [23].

Subjects’ characteristics were expressed as median and interquartile ranges. The Mann–Whitney U test was used to compare the two groups. Descriptive data were expressed as percentages. The statistical analysis was performed by R Studio (Version 1.1.463—© 2009–2018 RStudio, Inc., Boston, MA, USA). *p* values < 0.05 were considered statistically significant.

## 5. Results

Overall, 82 breath samples were obtained, but 14 of them were technically inadequate for the analysis and were eliminated. The chromatograms of the breath of the remaining 68 subjects, regarding 32 healthy controls (HC) and 36 patients with histologically proven colorectal cancer, were analyzed.

Patient characteristics are presented in Table 1. The two groups differed slightly in comorbidities and BMI. According to TNM classification, all CRC cases were M0; T and N stages and tumor location are reported in Table 2.

A description of the portable GC device and its operation are clarified in our previously published work [7,21,22].

**Figure 3:**
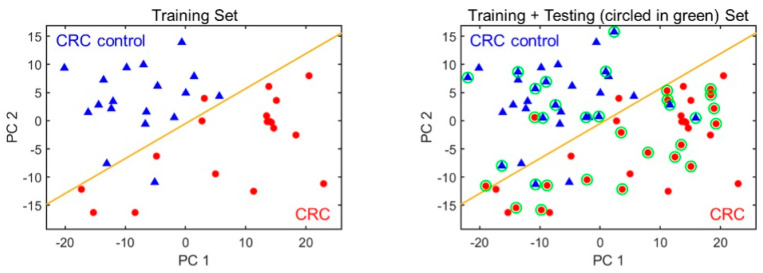
PCA plots using the three biomarkers for distinguishing CRC from non-CRC. (**Left**) The training set. (**Right**) Training plus testing set. CRC and non-CRC are denoted as red circles and blue triangles, respectively. The orange line marks the position of the boundary. The green circles indicate the test set data.

Among all peaks, only a few were associated with CRC. Of all compounds found, most are probably a result of normal metabolic activities, comorbidities or exogenous factors (use of drugs, air background, etc.) [24]. In order to select the most appropriate subset of peaks (i.e., biomarkers), 18 samples from colorectal cancer group and 18 healthy controls were used as the training set. The remaining 18 chromatograms from CRC group and 14 non-CRC were then used as the testing set. A set of three biomarker peak subsets were identified among approximately 100 VOCs detected, which yielded the overall classification accuracy of 91.2% with a sensitivity of 94.4% and a specificity of 87.5%. Using mass spectrometry, we preliminarily identified those 3 biomarkers as 2,4-dimethylhexane, 2,5-dimethylheptane and 2,2,5, 5-tetramethylhexane. The complete list of identified compounds in breath using the portable GC is shown in Table 3. The outlet of the portable GC was connected to an Agilent mass spectrometry (MS). The detailed setup and library used for compound identification is discussed in our previous paper (in Figure 3) [7]. Figure 3 shows the PCA plot of the training and the combined (training and testing) set. The corresponding statistics are given in Table 4.

## 6. Discussion

The improvement of screening tools for colorectal cancer still represents a major concern for health services in Western countries since the fecal occult blood test is hindered by inadequate accuracy and very low patients’ compliance. Therefore, several attempts are being made to find new non-invasive methodologies to detect CRC in the target population, including liquid biopsy [25], fecal DNA and fecal micro RNA [26,27,28].

Our study focused on the changes in the exhaled breath VOCs induced by CRC, using a new portable and easy-to-use device able to discriminate cancer patients from healthy controls by a machine learning approach. The preliminary results show that the three selected VOCs can identify colorectal cancer patients with a reassuring sensitivity of 94.4%, specificity of 87.5% and total accuracy of 91.2%. The biochemical pathways behind the significant difference in the distribution of these exhaled VOCs in CRC patients compared to non-cancer patients is still a matter of debate.

Several other investigations on the breath of CRC patients, including some from our group, have described different exhaled VOCs patterns with similar discriminatory ability, casting doubts on the reliability of this methodologic approach [29]. In fact, in 2012, our group reported a pattern of 15 compounds obtained after thermal-desorption and gas chromatography–mass spectrometry (GC-MS), demonstrating a discriminant performance with a sensitivity of 86%, a specificity of 83% and an accuracy of 85% [12]. Amal et al. identified four main VOCs able to identify advanced adenomas using GC-MS and a sensor analysis with a pattern recognition method [30]. In a further study from Markar, propanal was significantly elevated in the CRC cohort compared with control patients with sensitivity of 83.3% and specificity of 84.7% using ion flow tube mass spectrometry [31]. In the study carried out by Wang et al., solid-phase microextraction gas chromatography/mass spectrometry (SPME-GC/MS) was used to assess the exhaled VOCs. Eight VOCs were found to have higher levels in CRC patients compared to HC [32].

In a recent multicenter cross-sectional study from Zutphen, the Netherlands, an e-nose (Aeonose—the eNose Company) was used to perform 512 breath samplings. Patients affected by CRC or advanced adenomas with and without bowel preparation were recruited. Machine learning was applied to distinguish between breath profiles of controls and patients with (pre-)cancerous lesions. After a training model, a sensitivity of 95% and specificity of 64% for CRC and a sensitivity and specificity of 79% and 59% for advanced adenoma were reported [33].

Nevertheless, this multicenter study was limited by the high number of failed tests (up to 10%) and by some drawbacks of the e-nose technology including potential reproducibility issues, sensor drift, instrument variability and loss of sensitivity in the presence of alcohol and other compounds [34].

Steenhuis et al. investigated the feasibility of breath analysis as a tool for the follow-up of patients operated for CRC through an electronic nose confirming the pivotal role of VOCs in the evaluation of patient-disease status. In fact, the eNose identified extra luminal local recurrences or metastases of CRC with an overall accuracy of 0.81 [35].

In our recent study, we used an innovative breath sampler (ReCIVA^®^—Owlstone Medical, Cambridge, UK) able to select the alveolar air fraction, thus excluding environmental contamination. The breath was retained on Tenax tubes which were desorbed and analyzed by GC-MS. In a sample of 173 subjects (90 HC), fourteen VOCs were found to have significant discriminatory ability in discriminating between patients with and without colorectal cancer [20].

Furthermore, other studies have investigated the VOCs directly released by cancer tissue ex vivo, giving further information about the true source of these VOCs [36], in order to confirm that at least some of the VOCs identified in the breath of patients are effectively produced by the cancer tissue.

As far as we know, these three substances have never been reported before among the VOCs of interest in other studies on colorectal cancer. However, different groups may use different algorithms and therefore may obtain different markers.

This variability in the pattern of VOCs identified could be explained by the different sampling methodology, different analytic platforms and the chemical and statistical analysis used by different research groups.

However, all these studies demonstrated that the pattern of exhaled VOCs in cancer patients differs from HC and that this effect could be ascribed to the presence of cancer [37]. The true advantages of these techniques are the high patients’ compliance, together with the high sensitivity and specificity, representing an important step forward compared to the fecal occult blood immunochemical test, where the adherence of the target population to the screening program is less than 50% and the specificity of the test generally inadequate [38].

On the other hand, the main limitation of all these previous studies is the methodological complexity, the time and cost of the procedure and the impossibility of obtaining immediate feedback concerning the health status of each subject. All these drawbacks have been limited or eliminated using the new device of this study, where the fast analysis of the exhaled breath enables a rapid answer. Further development of this prototype is ongoing and, therefore, the cost of the device cannot be quantified at the moment. However, its easy and simple use, together with the reliability of the results, could facilitate its wide expansion to any center involved in colorectal cancer diagnosis.

The main limitations of this study are the small sample size, justified by the preliminary nature of this investigation, and the absence of a prospective blinding phase of the analysis, which is still ongoing.

The limited number of patients recruited did not allow for the evaluation of the sensitivity of the method according to the stage of the disease, although previous data from our group did not find a significant difference between stage I–II and stage III–IV [20]. Furthermore, no data are yet available on colorectal polyps, whose identification could allow primary colorectal prevention by endoscopic removal. In addition, our present portable GC can only separate and detect a limited range of VOCs in breath. Probable causes include the following:

1. Selection of Carbopack X and B.
2. Maximum working temperature of SSTI (250 °C).
3. Short column length.
4. Low maximum column operating temperature (120 °C).
5. Some compounds with ionization potential higher than 10.6 eV cannot be detected.

While these constraints did not prevent us from diagnosing, it would be possible to increase the separation and detection capability of the present portable GC to include additional VOCs. In addition, the present one-dimensional portable GC could be upgraded to a two-dimensional portable GC for better separation capability, as some of the peaks might be co-eluted in the present chromatograms. Sampling can be improved by reducing the sample time for faster breath collection. The bag can be replaced by TD tubes for sample collection for in-filed testing.

**Figure 4:**
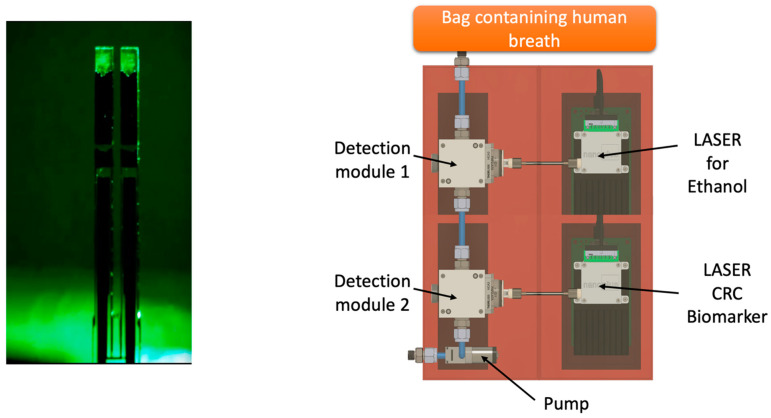
Future perspectives of QEPAS technology applied to the detection of VOCs exhaled from CRC patients.

Future perspectives will be oriented to develop a portable device able to identify the VOC of interest through specific sensors in order to get a digital output (YES/NO) with an immediate answer for the patient, like an alcohol test. One of the most promising technologies in this field is the QEPAS (quartz-enhanced photoacoustic laser spectroscopy) (Figure 4). As reported by Spagnolo et al., “*QEPAS is based on the absorption of modulated laser light by the target gas. The laser beam is focused between the prongs of a quartz tuning fork (QTF) at one of the antinode points of the QTF vibrational mode and is modulated at the associated resonance frequency or at one of its subharmonics. The energy of the excited roto-vibrational states is released* via *inelastic collisions among the surrounding molecules, generating a pressure wave. The pressure wave is detected by the quartz tuning fork, acting as a transducer of the prongs’ mechanical deflection induced by the pressure wave, into an electrical signal thanks to the piezoelectricity of the quartz*” [39,40,41].

In the future, the application of artificial intelligence coupled with machine learning could lead to remarkable advancements in the non-invasive early diagnosis and screening of CRC [42], exploring non-linear and complex relationships among features and providing insights into a “finer” choice of biomarkers [43,44].

## 7. Conclusions

These preliminary data show high sensitivity and specificity of the breath test in discriminating colorectal cancer patients from healthy subjects using a portable Micro-GC device. Further investigation on a larger cohort of patients is required to support these findings.

## Figures and Tables

**Table 1:** Subjects’ characteristics. Data are reported as percentage, median and interquartile ranges. * BMI: body mass index.

		CRC Group	HC Group	*p*
n		36	32	
Gender	Male	26 (72.20%)	19 (59.40%)	0.74
	Female	10 (27.80%)	13 (40.60%)	
Age		67 (64–77.25)	65 (54–68.75)	0.07
BMI *		27.50 (25.15–30.12)	25 (23.42–27.90)	0.03
Comorbidities	Cardiovascular	27 (75%)	11 (34.30%)	<0.01
	Pulmonary	6 (16%)	0	0.06
	Diabetes	7 (19.40%)	4 (12.50%)	0.40
Drugs	Antihypertensive	26 (72.20%)	9 (28.10%)	<0.01
	Antidiabetic	5 (13.80%)	4 (12.50%)	0.80
Weight Loss	yes	3 (8.30%)	1 (3.10%)	0.36
	no	33(91.60%)	31 (96.80%)	
Family history of CRC	yes	3 (8.30%)	2 (6.20%)	0.74
	no	33(91.60%)	30 (93.70%)	

**Table 2:** TNM classification, tumor location and median oncomarker levels.

Pathological T	T1	0
	T2	4 (11.10%)
	T3	27 (75%)
	T4	5 (13.80%)
Pathological N	N0	26 (72.20%)
	N1	7 (19.40%)
	N2	3 (8.30%)
Tumor location	Right	8 (22.20%)
	Transverse	2 (5.50%)
	Left	2 (5.50%)
	Sigmoid	16 (44.40%)
	Rectum	8 (22.20%)
CEA	ng/mL	2.8 (6.20–1.70)
CA 19.9	U/mL	9.6 (22.20–6.90)

**Table 3:** MS identified common peaks within human breath using portable GC.

Retention Time TR (s)	Library Compound	Retention Time TR (s)	Library Compound	Retention Time TR (s)	Library Compound
9	1,4-Dioxane-2,6-dione	260	1-Octene	470	Hexane, 2,2,5,5- tetramethyl-
38	Butane, 2-methyl-	266	Tetrachloroethylene	482	Heptane, 2,3,6-trimethyl-
42	Isoprene	267	4-Octene, (Z)-	502	a-Pinene
47	4-Penten-1-ol	271	4-Octene, (E)-	504	Cyclohexene, 4-methylene- 1-(1-methylethyl)-
53	Pentane, 2-methyl-	276	Octane	527	4-Octene, 2,6-dimethyl-, [S-(E)]-
62	1-Pentene, 2-methyl-	283	Heptane, 3,3-dimethyl-	533	2-Undecanethiol, 2-methyl-
65	n-Hexane	288	2-Heptene, 3-methyl-	558	Octane, 4-ethyl-
72	1-Pentanol, 2-methyl-	295	2-Octene	565	5-Ethyldecane
115	Hexane, 3-methyl-	304	Hexane, 2,3,5-trimethyl-	601	Decyl octyl ether
137	Heptane	316	Heptane, 2,4-dimethyl-	609	Decane, 2,6,7-trimethyl-
154	Cyclohexane, methyl-	327	Octane, 2-methyl-	616	Decane, 2,4,6-trimethyl-
158	1-Pentanol, 2-ethyl-4- methyl-	341	Heptane, 2,5-dimethyl-	624	Dodecane, 1-fluoro-
175	Pentane, 2,2,3-trimethyl-	362	Hexane, 2,3,4-trimethyl-	630	Decane, 2,2-dimethyl-
197	2,4-dimethylhexane	372	4,6-Octadiyn-3-one, 2- methyl-	665	2,2,7,7-Tetramethyloctane
209	Pentane, 2,3,4-trimethyl-	376	Heptane, 2,3-dimethyl-	656	Decane, 2,6,8-trimethyl-
214	Pentane, 2,3,3-trimethyl-	387	Octane, 4-methyl-	675	Decane, 2,5,9-trimethyl-
221	Hexane, 2,3-dimethyl-	394	Cyclopentane, 2-ethyl-1,1- dimethyl-	696	Heptane, 5-ethyl-2,2,3- trimethyl-
230	Hexane, 2,3-dimethyl-	412	Heptane, 2,2,4-trimethyl-	717	Decane, 2,6,7-trimethyl-
239	Heptane, 2,5-dimethyl-	422	Octane, 2,2-dimethyl-	735	Undecane, 3,6-dimethyl-
249	Hexane, 2,2,4-trimethyl-	441	Octane, 3,3-dimethyl-	768	Dodecane, 2,7,10- trimethyl-
255	Hexane, 2,2,5-trimethyl-				

**Table 4:** Statistics of breath analysis for CRC and non-CRC patients.

	Training Set			Testing Set			Training + Testing Set		
	CRC	Non-CRC	Total	CRC	Non-CRC	Total	CRC	Non-CRC	Total
Subject number	18	18	36	18	14	32	36	32	68
Positive	17	1	18	17	3	20	34	4	38
Negative	1	17	18	1	11	12	2	28	30
Specificity	94.4%			78.6%			87.5%		
Sensitivity	94.4%			94.5%			94.4%		
Positive predictive value	94.4%			85.0%			89.5%		
Negative predictive value	94.4%			91.7%			93.3%		
Total accuracy	94.4%			87.5%			91.2%		

## Data Availability

The data presented in this study are available on request from the corresponding author due to privacy and ethical reasons.

## References

[1] Arnold M., Sierra M.S., Laversanne M., Soerjomataram I., Jemal A., Bray F. (2017). Global patterns and trends in colorectal cancer incidence and mortality. Gut.

[2] Jideh B., Bourke M.J. (2018). Colorectal cancer screening reduces incidence, mortality and morbidity. Med. J. Aust..

[3] Kanth P., Inadomi J.M. (2021). Screening and prevention of colorectal cancer. BMJ.

[4] Li J.N., Yuan S.Y. (2019). Fecal occult blood test in colorectal cancer screening. J. Dig. Dis..

[5] Lauby-Secretan B., Vilahur N., Bianchini F., Guha N., Straif K., International Agency for Research on Cancer Handbook Working Group (2018). The IARC Perspective on Colorectal Cancer Screening. N. Engl. J. Med..

[6] Deng X., Nakamura Y. (2017). Cancer Precision Medicine: From Cancer Screening to Drug Selection and Personalized Immunotherapy. Trends Pharmacol. Sci..

[7] Sharma R., Zang W., Tabartehfarahani A., Lam A., Huang X., Sivakumar A.D., Thota C., Yang S., Dickson R.P., Sjoding M.W. (2023). Portable Breath-Based Volatile Organic Compound Monitoring for the Detection of COVID-19 During the Circulation of the SARS-CoV-2 Delta Variant and the Transition to the SARS-CoV-2 Omicron Variant. JAMA Netw. Open.

[8] Shekhawat J.K., Banerjee M. (2022). Role of Breath Biopsy in COVID-19. J. Appl. Lab. Med..

[9] Boots A.W., Bos L.D., van der Schee M.P., van Schooten F.J., Sterk P.J. (2015). Exhaled Molecular Fingerprinting in Diagnosis and Monitoring: Validating Volatile Promises. Trends Mol. Med..

[10] Bond A., Greenwood R., Lewis S., Corfe B., Sarkar S., O’Toole P., Rooney P., Burkitt M., Hold G., Probert C. (2019). Volatile organic compounds emitted from faeces as a biomarker for colorectal cancer. Aliment. Pharmacol. Ther..

[11] McFarlane M., Millard A., Hall H., Savage R., Constantinidou C., Arasaradnam R., Nwokolo C. (2019). Urinary volatile organic compounds and faecal microbiome profiles in colorectal cancer. Color. Dis..

[12] Altomare D.F., Di Lena M., Porcelli F., Trizio L., Travaglio E., Tutino M., Dragonieri S., Memeo V., de Gennaro G. (2013). Exhaled volatile organic compounds identify patients with colorectal cancer. Br. J. Surg..

[13] Wen Q., Boshier P., Myridakis A., Belluomo I., Hanna G.B. (2020). Urinary Volatile Organic Compound Analysis for the Diagnosis of Cancer: A Systematic Literature Review and Quality Assessment. Metabolites.

[14] Kneepkens C.M., Lepage G., Roy C.C. (1994). The potential of the hydrocarbon breath test as a measure of lipid peroxidation. Free Radic. Biol. Med..

[15] Fuchs P., Loeseken C., Schubert J.K., Miekisch W. (2010). Breath gas aldehydes as biomarkers of lung cancer. Int. J. Cancer.

[16] Phillips M., Cataneo R.N., Saunders C., Hope P., Schmitt P., Wai J. (2010). Volatile biomarkers in the breath of women with breast cancer. J. Breath Res..

[17] Abaffy T., Duncan R., Riemer D.D., Tietje O., Elgart G., Milikowski C., DeFazio R.A. (2010). Differential volatile signatures from skin, naevi and melanoma: A novel approach to detect a pathological process. PLoS ONE.

[18] Tsou P.H., Lin Z.L., Pan Y.C., Yang H.C., Chang C.J., Liang S.K., Wen Y.F., Chang C.H., Chang L.Y., Yu K.L. (2021). Exploring Volatile Organic Compounds in Breath for High-Accuracy Prediction of Lung Cancer. Cancers.

[19] Zhang J., Tian Y., Luo Z., Qian C., Li W., Duan Y. (2021). Breath volatile organic compound analysis: An emerging method for gastric cancer detection. J. Breath Res..

[20] Altomare D.F., Picciariello A., Rotelli M.T., De Fazio M., Aresta A., Zambonin C.G., Vincenti L., Trerotoli P., De Vietro N. (2020). Chemical signature of colorectal cancer: Case-control study for profiling the breath print. BJS Open.

[21] Zhou M., Sharma R., Zhu H., Li Z., Li J., Wang S., Bisco E., Massey J., Pennington A., Sjoding M. (2019). Rapid breath analysis for acute respiratory distress syndrome diagnostics using a portable two-dimensional gas chromatography device. Anal. Bioanal. Chem..

[22] Sharma R., Zang W., Zhou M., Schafer N., Begley L.A., Huang Y.J., Fan X. (2021). Real Time Breath Analysis Using Portable Gas Chromatography for Adult Asthma Phenotypes. Metabolites.

[23] Gillies C.E., Jennaro T.S., Puskarich M.A., Sharma R., Ward K.R., Fan X., Jones A.E., Stringer K.A. (2020). A Multilevel Bayesian Approach to Improve Effect Size Estimation in Regression Modeling of Metabolomics Data Utilizing Imputation with Uncertainty. Metabolites.

[24] Blanchet L., Smolinska A., Baranska A., Tigchelaar E., Swertz M., Zhernakova A., Dallinga J.W., Wijmenga C., van Schooten F.J. (2017). Factors that influence the volatile organic compound content in human breath. J. Breath Res..

[25] Allegretti M., Cottone G., Carboni F., Cotroneo E., Casini B., Giordani E., Amoreo C.A., Buglioni S., Diodoro M., Pescarmona E. (2020). Cross-sectional analysis of circulating tumor DNA in primary colorectal cancer at surgery and during post-surgery follow-up by liquid biopsy. J. Exp. Clin. Cancer Res..

[26] Loktionov A. (2020). Biomarkers for detecting colorectal cancer non-invasively: DNA, RNA or proteins?. World J. Gastrointest. Oncol..

[27] Pardini B., Ferrero G., Tarallo S., Gallo G., Francavilla A., Licheri N., Trompetto M., Clerico G., Senore C., Peyre S. (2023). A Fecal MicroRNA Signature by Small RNA Sequencing Accurately Distinguishes Colorectal Cancers: Results from a Multicenter Study. Gastroenterology.

[28] Xu F., Xu L., Wang M., An G., Feng G. (2015). The accuracy of circulating microRNA-21 in the diagnosis of colorectal cancer: A systematic review and meta-analysis. Color. Dis..

[29] Chung J., Akter S., Han S., Shin Y., Choi T.G., Kang I., Kim S.S. (2022). Diagnosis by Volatile Organic Compounds in Exhaled Breath in Exhaled Breath from Patients with Gastric and Colorectal Cancers. Int. J. Mol. Sci..

[30] Amal H., Leja M., Funka K., Lasina I., Skapars R., Sivins A., Ancans G., Kikuste I., Vanags A., Tolmanis I. (2016). Breath testing as potential colorectal cancer screening tool. Int. J. Cancer.

[31] Markar S.R., Chin S.T., Romano A., Wiggins T., Antonowicz S., Paraskeva P., Ziprin P., Darzi A., Hanna G.B. (2019). Breath Volatile Organic Compound Profiling of Colorectal Cancer Using Selected Ion Flow-tube Mass Spectrometry. Ann. Surg..

[32] Wang C., Ke C., Wang X., Chi C., Guo L., Luo S., Guo Z., Xu G., Zhang F., Li E. (2014). Noninvasive detection of colorectal cancer by analysis of exhaled breath. Anal. Bioanal. Chem..

[33] van Keulen K.E., Jansen M.E., Schrauwen R.W.M., Kolkman J.J., Siersema P.D. (2020). Volatile organic compounds in breath can serve as a non-invasive diagnostic biomarker for the detection of advanced adenomas and colorectal cancer. Aliment. Pharmacol. Ther..

[34] Wilson A.D. (2018). Application of Electronic-Nose Technologies and VOC-Biomarkers for the Noninvasive Early Diagnosis of Gastrointestinal Diseases (dagger). Sensors.

[35] Steenhuis E.G.M., Schoenaker I.J.H., de Groot J.W.B., Fiebrich H.B., de Graaf J.C., Brohet R.M., van Dijk J.D., van Westreenen H.L., Siersema P.D., de Vos Tot Nederveen Cappel W.H. (2020). Feasibility of volatile organic compound in breath analysis in the follow-up of colorectal cancer: A pilot study. Eur. J. Surg. Oncol..

[36] De Vietro N., Aresta A., Rotelli M.T., Zambonin C., Lippolis C., Picciariello A., Altomare D.F. (2020). Relationship between cancer tissue derived and exhaled volatile organic compound from colorectal cancer patients. Preliminary results. J. Pharm. Biomed. Anal..

[37] Altomare D.F., Di Lena M., Porcelli F., Travaglio E., Longobardi F., Tutino M., Depalma N., Tedesco G., Sardaro A., Memeo R. (2015). Effects of Curative Colorectal Cancer Surgery on Exhaled Volatile Organic Compounds and Potential Implications in Clinical Follow-up. Ann. Surg..

[38] Fisher D.A., Princic N., Miller-Wilson L.A., Wilson K., DeYoung K., Ozbay A.B., Limburg P. (2022). Adherence to fecal immunochemical test screening among adults at average risk for colorectal cancer. Int. J. Color. Dis..

[39] Elefante A., Menduni G., Rossmadl H., Mackowiak V., Giglio M., Sampaolo A., Patimisco P., Passaro V.M.N., Spagnolo V. (2020). Environmental Monitoring of Methane with Quartz-Enhanced Photoacoustic Spectroscopy Exploiting an Electronic Hygrometer to Compensate the H_2_O Influence on the Sensor Signal. Sensors.

[40] Patimisco P., Sampaolo A., Dong L., Tittel F.K., Spagnolo V. (2018). Recent advances in quartz enhanced photoacoustic sensing. Appl. Phys. Rev..

[41] Patimisco P., Scamarcio G., Tittel F.K., Spagnolo V. (2014). Quartz-enhanced photoacoustic spectroscopy: A review. Sensors.

[42] Viscaino M., Bustos J.T., Munoz P., Cheein C.A., Cheein F.A. (2021). Artificial intelligence for the early detection of colorectal cancer: A comprehensive review of its advantages and misconceptions. World J. Gastroenterol..

[43] Gallos I.K., Tryfonopoulos D., Shani G., Amditis A., Haick H., Dionysiou D.D. (2023). Advancing Colorectal Cancer Diagnosis with AI-Powered Breathomics: Navigating Challenges and Future Directions. Diagnostics.

[44] Mitsala A., Tsalikidis C., Pitiakoudis M., Simopoulos C., Tsaroucha A.K. (2021). Artificial Intelligence in Colorectal Cancer Screening, Diagnosis and Treatment. A New Era. Curr. Oncol..

